# Publisher Correction: Investigation of a Two-Patch Within-Host Model of Hepatitis B Viral Infection

**DOI:** 10.1007/s11538-026-01780-7

**Published:** 2026-10-17

**Authors:** Keoni Castellano, Omar Saucedo, Stanca M. Ciupe

**Affiliations:** 1 Department of Mathematics, Virginia Tech, 225 Stanger St., Blacksburg, 24060 VA United States; 2 Virginia Tech Center for the Mathematics of Biosystems, Virginia Tech, 765 West Campus Drive, Blacksburg, 24060 VA United States

**Correction to: Bulletin of Mathematical Biology (2025) 87:181** https://doi.org/10.1007/s11538-025-01555-6

Due to a typesetting error the author names are incorrect. The correct author names are: Keoni Castellano, Omar Saucedo, and Stanca M. Ciupe.

